# Smoke-Free Rules in Homes and Cars Among Smokers and Nonsmokers in Minnesota

**DOI:** 10.5888/pcd15.170355

**Published:** 2018-03-15

**Authors:** Michael J. Parks, John H. Kingsbury, Raymond G. Boyle, Sharrilyn Evered

**Affiliations:** 1 Department of Pediatrics, Medical School, University of Minnesota, St Paul, Minnesota; 2 Health Promotion and Chronic Disease Division, Minnesota Department of Health, St Paul, Minnesota; 3 Office of Statewide Health Improvement Initiative, Minnesota Department of Health, St Paul, Minnesota; 4 Department of Research Programs, ClearWay Minnesota, Minneapolis, Minnesota; 5 Center for Health Statistics, Minnesota Department of Health, St Paul, Minnesota

## Abstract

We examined prevalence and predictors of comprehensive smoke-free household rules (ie, smoke-free homes and cars) among smokers and nonsmokers in Minnesota. Data came from the 2014 Minnesota Adult Tobacco Survey; weighted analyses consisted of descriptive analyses and multivariate logistic regression analyses. Most adult smokers implemented home-only smoke-free rules (43%) while most nonsmokers implemented comprehensive smoke-free rules (home and car; 85%). Comprehensive smoke-free rules were more common among people with high socioeconomic status (SES), married people, and people who did not live with a smoker; those with a child in the home were more likely to implement smoke-free homes but not smoke-free cars. Public health practitioners should focus on addressing the majority of smokers who do not implement comprehensive smoke-free household rules, such as low-SES populations, and addressing caregivers who do not implement smoke-free car rules.

## Objective

Smoke-free public policies reduce secondhand smoke in public places; however, private spaces, such as the home and car, are common locations for secondhand smoke exposure (1). Voluntary smoke-free rules in homes can reduce secondhand smoke and tobacco use, especially among households with children (2–4). Smoke-free homes have become more prevalent, but disparities persist (2,5–7). Limited information exists on comprehensive smoke-free rules (ie, smoke-free homes and cars) (2,7,8), which are optimal, particularly for children (2,9). We examined prevalence and predictors of smoke-free rules among smokers and nonsmokers in Minnesota.

## Methods

Data came from the 2014 Minnesota Adult Tobacco Survey (MATS). MATS is a statewide, cross-sectional landline and cellular telephone survey of 9,304 Minnesotans aged 18 years or older, which yielded a combined response rate of 71%. MATS was approved by Minnesota Department of Health’s Institutional Review Board. Survey weights accounted for sampling and ensured statewide representativeness. Smoke-free rules were measured with 2 questions: 1) “Which statement best describes rules about smoking inside your home (excluding porches and garages): not allowed anywhere, allowed some places or at some times, or allowed anywhere”; and 2) “In the vehicles (excluding motorcycles) that you or your family who live with you own or lease, is smoking . . . allowed, sometimes allowed in at least one vehicle, or never allowed in any vehicle.” A composite, 4-category variable captured smoke-free rules: comprehensive (smoke-free home and car), home-only (smoke-free home, not car), car-only (smoke-free car, not home), and no rules (smoking allowed in home and car). Covariates were age (4 categories), education (4 categories), low-income (yes or no), race/ethnicity (white or other), sex (male or female), marital status (married or not married), living with a child aged less than 18 years (yes or no), location (metropolitan county or nonmetropolitan county), and living with a smoker (yes or no). We used a 3-category measure of smoking intensity: light, moderate, and heavy.

Descriptive analyses were used to compare demographic characteristics and smoking behaviors across different household smoke-free rules for smokers and nonsmokers separately. All analyses were conducted using the *svyset* command in Stata, version 13 (StataCorp LLC). Stata’s default of *F* ratios and adjusted Wald tests were used to compare continuous variables across smoke-free rules categories; χ^2^ statistics and design-adjusted *F* ratios were used to compare binary variables. A multivariate logistic regression was used to assess characteristics associated with comprehensive smoke-free rules for smokers and nonsmokers separately.

## Results

Most adult smokers implemented home-only smoke-free rules (43%) or did not implement any smoke-free rules (31%) (Figure). A smaller proportion of smokers implemented comprehensive smoke-free rules (home and car) (19%). Most nonsmokers implemented comprehensive smoke-free rules (85%) (Figure). There were significant differences in demographics and behaviors across different smoke-free rules among smokers and nonsmokers (Table).

**Figure:**
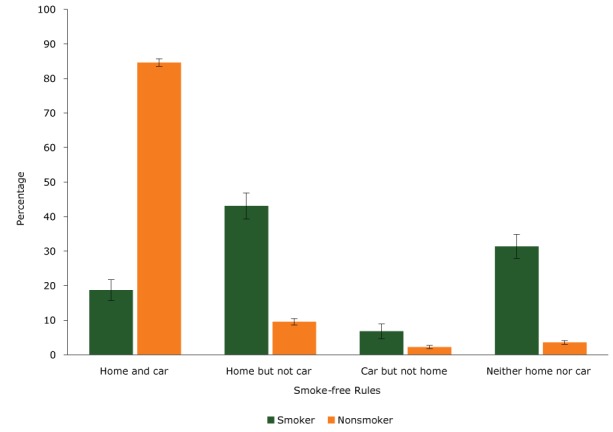
Percentage of smokers and nonsmokers who implemented voluntary smoke-free rules in the home and car, Minnesota Adult Tobacco Survey, 2014. Error bars indicate 95% confidence intervals. **Table:** 

Category	Smoker, % (95% Confidence Interval)	Nonsmoker, % (95% Confidence Interval)
Home and car	19 (16–22)	85 (84–86)
Home but not car	43 (39–47)	10 (9–11)
Car but not home	7 (5–9)	2 (2–3)
Neither home nor car	31 (28–35)	4 (3–4)

**Table:** Smoke-Free Rules in the Home and Car and Comparative Characteristics Among Minnesota Adult Smokers and Nonsmokers, Minnesota Adult Tobacco Survey, 2014^a^

Variable^b^	Home and Car	Home But Not Car	Car But Not Home	Neither Home Nor Car	*P* Value^c^
**Smokers**					
**Age, y**					
18–24	24.6 (0.34)	45.4 (0.39)	04.0 (0.15)	26.0 (0.35)	<.001
25–44	19.5 (0.32)	54.7 (0.40)	03.0 (0.14)	22.9 (0.34)	<.001
45–64	16.3 (0.38)	32.3 (0.48)	12.5 (0.34)	38.9 (0.50)	<.001
≥65	16.9 (0.50)	13.6 (0.46)	10.0 (0.40)	59.7 (0.66)	<.001
**White (vs other)**	17.9 (0.37)	44.2 (0.47)	05.3 (0.21)	32.5 (0.45)	.03
**Male (vs female)**	19.5 (0.35)	44.1 (0.44)	05.6 (0.20)	30.8 (0.41)	.57
**Metropolitan county (vs nonmetropolitan county)**	20.8 (0.32)	42.8 (0.39)	08.3 (0.22)	28.1 (0.35)	.03
**Education^d^, category mean (SD)**	02.6 (0.78)	02.6 (0.72)	02.1 (0.70)	02.3 (0.86)	<.001
**Low income^e^ (vs other)**	12.0 (0.32)	33.5 (0.47)	09.9 (0.30)	44.6 (0.50)	<.001
**Married (vs unmarried)**	22.5 (0.38)	43.8 (0.45)	05.9 (0.21)	27.8 (0.41)	.18
**Child aged <18 y in household**	21.1 (0.34)	53.8 (0.41)	01.9 (0.12)	23.2 (0.35)	<.001
**Lives with smoker**	14.7 (0.31)	40.1 (0.43)	08.2 (0.24)	36.9 (0.42)	<.01
**Smoking intensity^f^**					
Light	25.7 (0.40)	45.9 (0.45)	06.8 (0.23)	21.6 (0.37)	<.001
Moderate	05.9 (0.22)	39.7 (0.47)	06.9 (0.24)	47.5 (0.48)	<.001
Heavy	06.0 (0.25)	36.1 (0.51)	07.6 (0.28)	50.3 (0.53)	<.001
**Nonsmokers**					
**Age, y**					
18–24	71.5 (0.35)	21.6 (0.32)	02.9 (0.13)	04.0 (0.15)	<.001
25–44	87.4 (0.28)	09.5 (0.25)	01.6 (0.10)	01.5 (0.10)	<.001
45–64	84.8 (0.37)	07.9 (0.28)	02.1 (0.15)	05.2 (0.23)	<.001
≥65	87.4 (0.44)	05.1 (0.29)	03.5 (0.24)	04.0 (0.26)	<.001
**White (vs other)**	85.0 (0.37)	09.3 (0.30)	02.1 (0.15)	03.6 (0.19)	.21
**Male (vs female)**	82.9 (0.36)	10.6 (0.30)	02.3 (0.15)	04.2 (0.19)	.02
**Metropolitan county (vs nonmetropolitan county)**	84.5 (0.31)	09.7 (0.26)	02.3 (0.13)	03.5 (0.16)	.86
**Education^d^, category mean (SD)**	03.0 (0.91)	02.8 (0.76)	02.7 (1.10)	02.6 (0.82)	<.001
**Low income^e^ (vs other)**	77.6 (0.01)	13.4 (0.01)	03.1 (0.01)	05.8 (0.01)	<.001
**Married (vs unmarried)**	88.9 (0.01)	06.4 (0.01)	01.8 (0.00)	02.9 (0.00)	<.001
**Child aged <18 y in household**	87.0 (0.01)	09.9 (0.01)	01.1 (0.00)	02.0 (0.00)	<.001
**Lives with smoker**	44.2 (0.02)	34.8 (0.02)	04.1 (0.01)	16.9 (0.02)	<.001

a All estimates were derived by using survey weights.

b All values are % (SD) except where otherwise noted.

c *F* ratios and adjusted Wald tests were used to compare continuous variables; χ^2^ statistics and design-adjusted *F* ratios were used to compare binary variables.

d Education was a 4-category measure: 1 = no high school degree; 2 = high school degree; 3 = more than high school degree but no college degree; and 4 = college degree or more.

e Low income was defined as the lowest 25% of the distribution of our income measure, which was equivalent to less than $30,000 total household income per year.

f Smoking intensity is based on smoking frequency and number of cigarettes per day (light = <15 cigarettes per day and both daily and nondaily smoker; moderate = >15 but <25 cigarettes per day and a daily smoker; heavy = >25 cigarettes per day and a daily smoker.

Multivariate logistic regressions showed that income, marital status, and smoking intensity were related to comprehensive smoke-free rules among smokers. The odds of implementing comprehensive rules decreased for moderate and heavy smokers compared with light smokers (adjusted odds ratio [AOR] for moderate smokers, 0.13; 95% CI, 0.07–0.23; and AOR for heavy smokers, 0.21; 95% CI, 0.06–0.71). The odds of implementing comprehensive rules increased for married smokers compared with unmarried smokers (AOR, 1.80; 95% CI, 1.09–2.98). Low-income status was negatively associated with implementing comprehensive rules (AOR, 0.57; 95% CI, 0.34–0.94). Having a child in the home aged less than 18 years had a nonsignificant association with comprehensive rules for smokers (supplementary analyses showed a significant association with smoke-free home rules).

For nonsmokers, men were less likely than women to implement comprehensive smoke-free rules (AOR, 0.75; 95% CI, 0.61–0.92). Higher education levels were positively related to comprehensive rule implementation (AOR, 1.30; 95% CI, 1.15–1.48), and low-income status was negatively related to comprehensive rule implementation (AOR, 0.75; 95% CI, 0.59–0.97). Married nonsmokers were more likely to implement comprehensive rules than were unmarried nonsmokers (AOR, 1.98; 95% CI, 1.58–2.50), and the odds of implementing comprehensive rules were lower for nonsmokers who lived with a smoker than with those who did not (AOR, 0.08; 95% CI, 0.07–0.11). Having a child in the home under 18 years had a nonsignificant association with implementing comprehensive smoke-free rules for nonsmokers.

## Discussion

This study adds to the literature on voluntary, smoke-free rules by establishing the prevalence and predictors of comprehensive smoke-free rules (ie, in homes and cars). We found marked disparities in comprehensive smoke-free rule implementation across smoking status and key demographics.

Although 85% of nonsmokers implemented comprehensive rules, only 19% of smokers implemented such comprehensive rules, and 43% implemented home-only rules. Previous research has documented an increase in smoke-free rules in homes (2), indicating that the general public understands the harm of secondhand smoke in homes. However, we found that smoking in the car was common, even among smokers who implement a smoke-free home.

People with low income and low education levels were less likely to implement comprehensive smoke-free rules. Practitioners should focus on reducing SES-related barriers to implementing comprehensive smoke-free rules (eg, health care access, “knowledge gaps” of the danger of secondhand smoke). Tailored health education efforts regarding comprehensive smoke-free rules may help to address these disparities. Living with a smoker was also a barrier to implementation of smoke-free rules (among smokers and nonsmokers); consequently, programs should communicate the importance of comprehensive smoke-free rules by addressing the entire household.

Living with a child under age 18 years predicted smoke-free home rules, supporting previous research (2,10), but living with a child did not predict smoke-free car rules for most smokers. Private spaces, such as homes and cars, are major sources of secondhand smoke exposure, particularly for children (1). Implementing less than comprehensive smoke-free rules increases the risk of secondhand smoke exposure, and voluntary rules can protect children against secondhand smoke in private spaces that are typically unaffected by public policy (3,11). Given the dangers associated with secondhand smoke exposure in confined spaces such as cars (12), public health programs, media campaigns, and primary care interventions should promote comprehensive smoke-free rules among smokers, and particularly among caregivers and parents who implement less than comprehensive rules (3,9).
